# Safety & efficacy of a robotic hip exoskeleton on outpatient stroke rehabilitation

**DOI:** 10.1186/s12984-024-01421-x

**Published:** 2024-07-30

**Authors:** Rebecca Macaluso, Matt Giffhorn, Sara Prokup, Brice Cleland, Jusuk Lee, Bokman Lim, Minhyung Lee, Hwang-Jae Lee, Sangeetha Madhavan, Arun Jayaraman

**Affiliations:** 1 Max Nader Lab for Rehabilitation Technologies and Outcomes Research, Shirley Ryan AbilityLab, 355 E Erie St., Chicago, IL 60611 USA; 2 College of Applied Health Sciences, University of Illinois, Chicago, USA; 3 Department of Radiology and Biomedical Imaging, University of California, San Francisco, USA; 4 Robot R&D Team, WIRobotics Inc, Yongin, Republic of Korea; 5 Robot System Team, Samsung Research, Samsung Electronics, Suwon, Republic of Korea; 6 Robot Business Team, Samsung Electronics, Suwon, Republic of Korea; 7 Department of Physical Medicine and Rehabilitation, Northwestern University, 710 N Lake Shore Dr, Chicago, IL 60611 USA

**Keywords:** Stroke rehabilitation, Exoskeleton, Gait training, Outpatient

## Abstract

**Objective:**

The objective of this study was to analyze the safety and efficacy of using a robotic hip exoskeleton designed by Samsung Electronics Co., Ltd., Korea, called the Gait Enhancing and Motivating System-Hip (GEMS-H), in assistance mode only with the poststroke population in an outpatient-rehabilitation setting.

**Methods:**

Forty-one participants with an average age of 60 and average stroke latency of 6.5 years completed this prospective, single arm, interventional, longitudinal study during the COVID-19 pandemic. Significant modifications to the traditional outpatient clinical environment were made to adhere to organizational physical distancing policies as well as guidelines from the Centers for Disease Control. All participants received gait training with the GEMS-H in assistance mode for 18 training sessions over the course of 6–8 weeks. Performance-based and self-reported clinical outcomes were assessed at four time points: baseline, midpoint (after 9 training sessions), post (after 18 training sessions), and 1-month follow up. Daily step count was also collected throughout the duration of the study using an ankle-worn actigraphy device. Additionally, corticomotor excitability was measured at baseline and post for 4 bilateral lower limb muscles using transcranial magnetic stimulation.

**Results:**

By the end of the training program, the primary outcome, walking speed, improved by 0.13 m/s (*p* < 0.001). Secondary outcomes of walking endurance, balance, and functional gait also improved as measured by the 6-Minute Walk Test (47 m, *p* < 0.001), Berg Balance Scale (2.93 points, *p* < 0.001), and Functional Gait Assessment (1.80 points, *p* < 0.001). Daily step count significantly improved with and average increase of 1,750 steps per day (*p* < 0.001). There was a 35% increase in detectable lower limb motor evoked potentials and a significant decrease in the active motor threshold in the medial gastrocnemius (-5.7, *p* < 0.05) after training with the device.

**Conclusions:**

Gait training with the GEMS-H exoskeleton showed significant improvements in walking speed, walking endurance, and balance in persons with chronic stroke. Day-to-day activity also improved as evidenced by increased daily step count. Additionally, corticomotor excitability changes suggest that training with this device may help correct interhemispheric imbalance typically seen after stroke.

**Trial Registration:**

This study is registered with ClinicalTrials.gov (NCT04285060).

## Introduction

Stroke is the leading cause of adult-onset disability in the United States. Up to 80% of stroke survivors experience considerable gait impairments, such as reduced walking speeds, reduced endurance, and asymmetrical walking patterns, resulting in limited capacity for community ambulation [1]. These mobility deficits are the result of a combination of numerous neuromuscular changes post stroke, including: reduced corticospinal drive and control [2], muscle atrophy and weakness [3], impaired balance and postural control [4], and abnormal muscle synergies [5].

The goal of post-stroke rehabilitation is to facilitate return to an individual’s highest level of function for employment and social and community participation [6]. The return of mobility and walking is a crucial part of this return to everyday function [7]. There is strong evidence that individuals who have had a stroke continue to recover years after the original neurological insult [8, 9]. Thus, continuing therapy as part of outpatient care or in home/community settings provides the opportunity for individuals with chronic stroke to continue to recover walking function. One group of technologies that shows promise in seamless integration with the outpatient and community settings are unconstrained, light-weight, modular, robotic exoskeletons. The use of these modular exoskeletons may allow intense gait training to be combined with activities of daily living. Furthermore, these robots can also target specific chronic impairments without sacrificing the functional task practice. However, there are a limited number of studies that investigate the impact of this technology on walking performance in the chronic stroke population in the outpatient, home, and/or community settings [10, 11]. More clinical studies are warranted to help provide evidence to guide this new generation of light-weight, modular robots to become part of everyday rehabilitation strategies.

The primary objective of this study was to analyze the safety and efficacy, as measured by clinical outcomes, daily step count, and corticomotor excitability, of using the Samsung Gait Enhancing and Motivating System-Hip (GEMS-H) in assistance mode with the poststroke population as part of an outpatient-rehabilitation program. The primary hypothesis was that subjects would demonstrate improved clinical outcomes, as well as higher daily step counts and increased corticomotor excitability, after completing 18 training sessions.

## Methods

### Participants

Trial participants were recruited between February 2020 and April 2021 from the Shirley Ryan AbilityLab (Chicago, IL, USA). Inclusion criteria for the trial were: age 18–85 years; at least 30-days post stroke; Mini-Mental State Examination score > 17; initial walking speed of 0.4–0.8 m/s; ability to walk at least 10 m with no more than 1 person assisting; ability to safely fit into device specifications and tolerate minimum assistance; and physician approval for patient participation. Individuals were excluded from the study if they had: major orthopedic surgery (i.e., hip, knee, and/or ankle joint replacement) within the last 3 months; cardiac bypass or valve procedure within the last 6 months; any other serious cardiac conditions within the last 3 months; severe osteoporosis as indicated by physician medical clearance; pregnancy; uncontrolled hypertension; lower extremity fracture; pre-existing neurological disorders; history of major head trauma, lower extremity amputation, non-healing ulcers of a lower extremity, ongoing active infections, legal blindness, or a history of significant psychiatric illness; or current participation in any other program (i.e., structured outpatient therapy, home health physical therapy, or another clinical trial). Walking speed criteria was selected to target limited community ambulators as defined by Perry et al. [12]. Subjects that were enrolled in the study were excluded from the corticomotor excitability protocol if they had: pacemakers; metal implants in the head region; history of unexplained, recurring headaches, epilepsy/seizures/skull fractures or skull deficits; medications that lower seizure threshold; or history of concussion in the last 6 months. Those that qualified were also given the opportunity to opt-out of the corticomotor excitability portion without having to withdraw from the study.

**Fig. 1:**
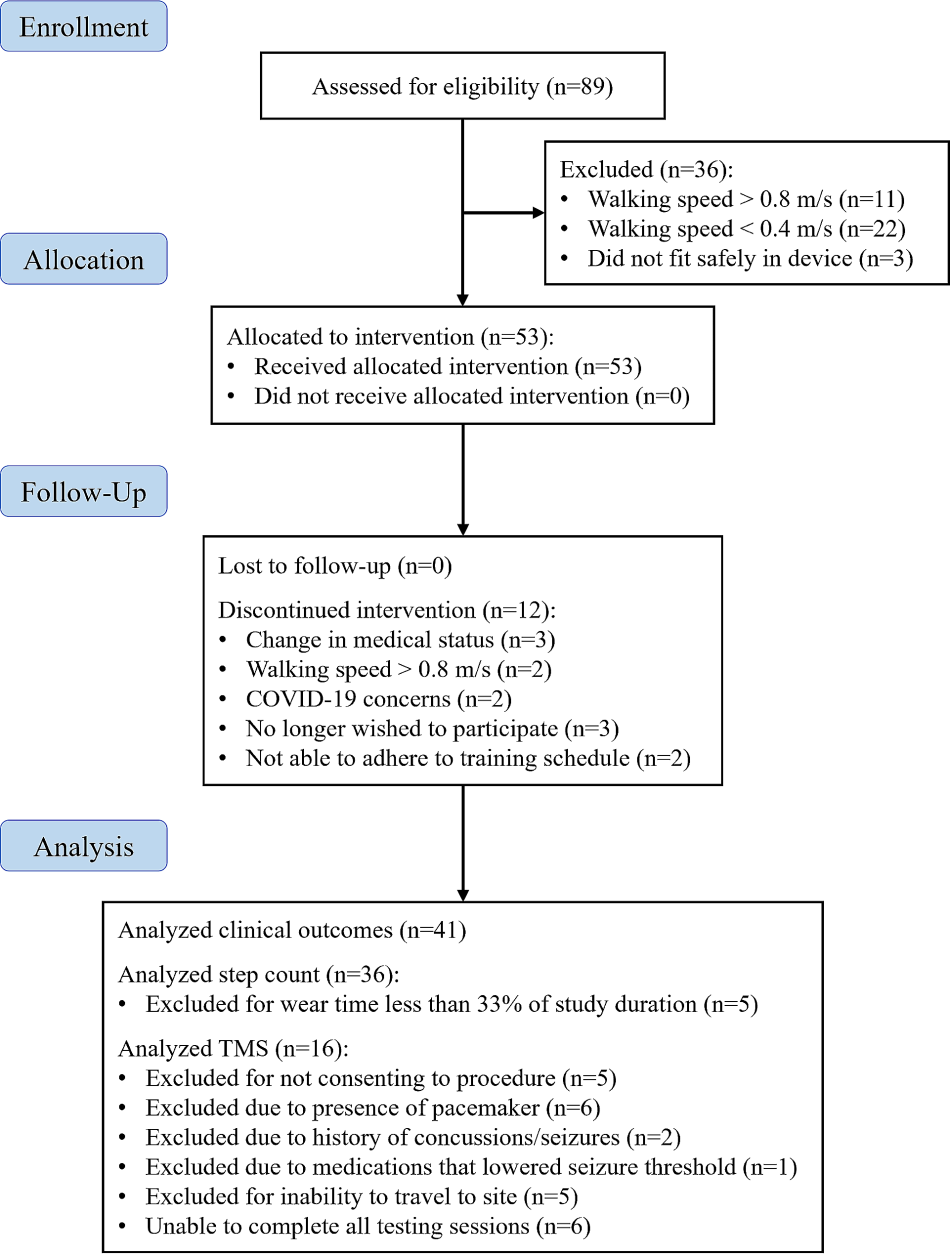
Diagram of subject disposition throughout intervention. Reasons for not meeting inclusion criteria or not completing the protocol are also provided

Of the 89 potential participants screened, 53 qualified for the study and 41 completed the intervention training program (Fig. 1). Demographic information for the 41 participants who completed training is reported in Table 1. Most participants were in the chronic phase of stroke recovery [37 (90%) vs. 4 (10%) sub-acute] with an average latency of 6.51 ± 6.21 years.

**Table 1:** Demographics of participants

Characteristics	Values
Age in years, mean (SD)	60 (9)
Sex, male, n (%)	22 (54)
Years since stroke, mean (SD)	6.5 (6.2)
Hemiparesis, left, n (%)	24 (59)
Type of stroke	
Hemorrhagic, n (%)	14 (34)
Ischemic, n (%)	23 (56)
Unknown/Not reported, n (%)	4 (10)
Assistive device used	
Single-point cane, n (%)	12 (29)
Large-based quad cane, n (%)	1 (2)
Small-based quad cane, n (%)	8 (20)
Rollator walker, n (%)	2 (5)
None, n (%)	18 (44)
Orthotic used	
Ankle foot orthosis (AFO), n (%)	18 (44)
Articulated AFO, n (%)	3 (7)
Carbon fiber AFO, n (%)	2 (5)
Active ankle, n (%)	1 (2)
None, n (%)	17 (41)

Abbreviations: AFO – ankle foot orthosis

### Standard protocol approvals, registration, and patient consents

This study was approved by the Institutional Review Board of Northwestern University (Chicago, IL, USA). All participants provided written, informed consent. This study was registered with ClinicalTrials.gov under: NCT04285060.

#### Interventions

All eligible study participants were enrolled in a single group and received gait training while wearing the Samsung GEMS-H device in assistance mode. The intervention consisted of therapist-guided gait training 2–4 times per week for an average duration of 6–8 weeks (18 sessions total). The number of sessions was chosen to match Medicare reimbursement guidelines for standard outpatient stroke rehabilitation. All training sessions were administered by a trained and licensed physical therapist in the outpatient clinic at a rehabilitation hospital.

Each training session consisted of 45 min of gait training while wearing the GEMS-H in assistance mode. A minimum of 30 min (2 units) was spent doing task specific gait training (rating of perceived exertion 13–17), followed by an additional 15 min (1 unit) focused on patient specific goals. When appropriate, this time was used for additional walking training.

During training sessions, participants were allowed to use assistive devices (AD) or prescribed orthotics as needed to ensure safety. When determined to be appropriate and safe, participants were allowed to train without their assistive devices to provide a challenge. For assessment sessions, participants’ use of orthotics and assistive devices was kept consistent both with and without the GEMS-H. This was also kept consistent across timepoints. If a participant progressed beyond either the type of bracing or assistive device used, both the initial and current bracing/AD were used during testing at subsequent evaluation timepoints. Of the 41 participants, 23 (56%) used an assistive device and 24 (59%) used a lower limb orthosis. A breakdown of the types of assistive devices and orthotics used can be found in Table 1.

#### GEMS-H device

**Fig. 2:**
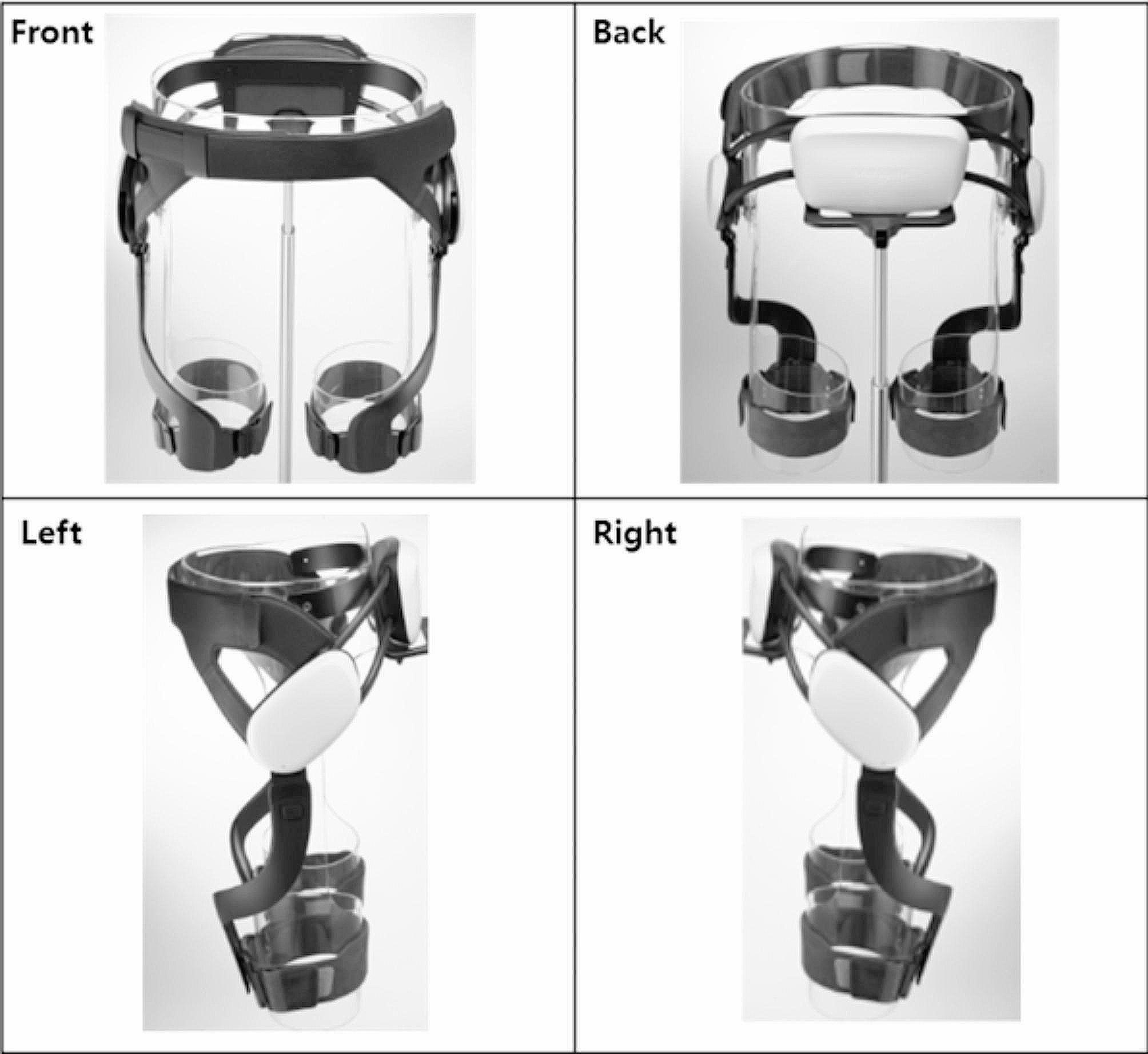
The Gait Enhancing and Motivating System-Hip (GEMS-H) from the front, back, left, and right sides

The GEMS-H is a hip-based, robotic exoskeleton worn around the waist and fastened to the thighs to aid in hip flexion and extension (Fig. 2). It comes in three sizes and the exact width of each size can be adjusted to fit an individual’s body. The device has a pair of actuators that generate assistive forces (i.e., torque) at each hip joint. The GEMS-H is controlled through a custom-built application on a hand-held tablet. Through the application, a therapist can turn the assistance on or off as well as modify the amount of assistance by adjusting gain and delay parameters. Gain refers to the amplitude of torque output by the motors at the hip, increasing or decreasing the amount of assistance provided with a maximum value of 15 (equivalent to 12 Nm). Delay refers to the amount of time the controller should wait before signaling the motors to provide assistance. This allows the timing of assistance to be tuned based on the walking speed of the wearer, with a range of 0.15–0.25 s. For an in-depth explanation on operation of the GEMS-H and its control algorithm, please refer to papers [13–16]. During training, the therapist would adjust both the gain and delay parameters based on the subject’s walking speed and rate of perceived effort. This ensured that the device could adapt to subject improvements while maintaining challenging training sessions throughout the intervention.

### Clinical assessments

Participants were assessed at 4 time points: baseline (Pre), after 9 therapy sessions (Mid), after 18 therapy sessions (Post), and 1-month follow up after the last therapy session (1MFU). All assessments were completed by research personnel that were also licensed physical therapists.

Each assessment consisted of performance-based measures as well as patient reported outcomes. Performance measures included the 10-Meter Walk Test (10MWT) for self-selected comfortable and fastest safe walking speeds (SSV/FV) [17], 6-Minute Walk Test (6MWT) [18], Berg Balance Scale (BBS) [19], Functional Gait Assessment (FGA) [20], 5 Times Sit-to-Stand Test (5xSTS) [21], and the Fugl-Meyer Assessment (FMA) [22]. Patient reported measures included the Modified Falls Efficacy Scale (mFES) [23], Activities-specific and Balance Confidence Scale (ABC) [24], Stroke Impact Scale (SIS) [25], Patient Health Questionnaire-9 (PHQ-9) [26], and Stroke Specific Quality of Life (SSQoL) [27].

To assess clinical impact, changes in self-selected comfortable walking speed (10MWT-SSV), walking endurance (6MWT), and balance (BBS, FGA) were compared to a minimally clinically important difference (MCID). If an MCID was unavailable, the minimal detectable change (MDC) was used. For walking speed and endurance, the MCID is 0.14 m/s and 50 m, respectively [28]. For balance measures, the MDC of the BBS is 4.13 points [29], while the MDC for the FGA is 4.2 points [30].

### Safety and immediate mobility assistance

Device safety was assessed by the number of device-related adverse events, including serious adverse events and falls, that occurred from baseline through the one-month follow-up. The threshold for the GEMS-H to be considered safe was a device-related adverse event rate less than 5%. To assess whether the device could lead to improved walking performance simply upon donning (i.e., immediate mobility assistance), participants completed the 10MWT and 6MWT both with and without the GEMS-H. All other performance measures were only completed without the device.

### Daily stepping activity

Once enrolled in the study, each subject was issued an activity monitor (ActiGraph wGT3X-BT, ActiGraph LLC., Pensacola, FL, USA) and asked to wear it on their non-paretic ankle during waking hours. Participants wore the ActiGraph during training sessions, but it was removed to charge and download data during assessment sessions. ActiGraphs were collected and final data was downloaded at the one-month follow-up. When worn, the ActiGraph collected triaxial accelerometer data at 30 Hz. Wear time validation was performed using ActiLife (ActiLife 6.13.4) following the algorithm developed by Choi et al. [31]. Daily step count was then estimated using calculations from Freedson et al. [32].

### Corticomotor excitability

To assess corticomotor excitability for each muscle, qualifying participants were evaluated at Pre and Post using a single-pulse transcranial magnetic stimulator (TMS) (Magstim 200, Magstim Inc., MN, USA). Muscle activity was recorded bilaterally for the tibialis anterior (TA), medial gastrocnemius (MG), rectus femoris (RF), and biceps femoris (BF) (Bagnoli 8, Delsys, MA, USA; frequency: 2000 Hz, gain: 1000, bandpass filter: 20–450 Hz) and sampled with Spike2 (Cambridge Electronic Design, Cambridge, UK). Electromyography (EMG) preparation and placement followed SENIAM recommendations [33].

All TMS stimuli were applied through a double-cone coil with posterior-to-anterior current flow at a maximal frequency of 0.25 Hz [34]. Prior to stimulation, participants performed three maximal voluntary isometric contractions (MVICs) for each muscle, and the largest rectified EMG amplitude was recorded. The optimal position for TMS (hotspot) for each hemisphere was determined by systematically moving the coil while participants performed unilateral, isometric contractions of the contralateral TA at 10% of MVIC [35]. The coil was initially positioned over a spot that approximated the lower limb motor cortex (1 cm posterior and 1 cm lateral to the vertex), and then was moved in small spatial increments. The hotspot was then identified as the position eliciting the largest, consistent motor evoked potential (MEP) in the target muscle at the lowest stimulator intensity. The hotspot for each hemisphere was used for all muscles of the contralateral limb because of the proximity and overlap of the motor representations of lower limb muscles [36].

The active motor threshold (AMT) of each muscle was determined as the minimum stimulus needed to elicit a contralateral MEP with a peak-to-peak amplitude greater than or equal to 0.1 mV in 5 out of 10 trials [37] during unilateral isometric contractions of the contralateral TA at 10% of MVIC. Subsequently, 15 stimuli at 130% of AMT were applied to elicit contralateral MEPs in each muscle. In cases where no MEPs were detected or 130% of AMT was above the maximal stimulator output, 15 stimuli were applied at the highest intensity that did not elicit an obscuring stimulus artifact. The hotspot and AMT for each leg of each participant was maintained within sessions and re-determined between sessions.

The root mean squared (RMS) of the EMG from MEP onset to offset was averaged across all stimuli and normalized to the background RMS present 50 milliseconds prior to stimulation [34]. The MEP latency and duration were also assessed [38]. To quantify intracortical inhibition, we measured the duration of the silent period [39].

### Statistical analyses

We implemented a repeated-measure, single arm design. Statistical analyses were performed using R (Version 4.1.1) (R Foundation for Statistical Computing, Vienna, Austria) with a significance level of 0.05 unless otherwise noted. Clinical measures were analyzed across all four time points, while daily step count and corticomotor excitability were analyzed for two of the four time points (Pre vs. Post).

Separate generalized, linear, mixed-effects models were used to examine changes in performance based clinical outcome measures over time. Each outcome was treated as a dependent variable while time point (Pre, Mid, Post, 1MFU), number of years since stroke, and stroke type were treated as fixed effects. To allow more flexibility, a subject-specific random intercept was included with each model. Post hoc tests determined whether differences in time points were statistically significant, adjusting for repeated measures using the Bonferroni correction.

For daily step activity and corticomotor excitability, subjects were removed from analysis if Pre or Post training data points were missing. For daily step activity, participants were also removed if a wear time validation revealed wear time less than 33% of the study duration. Paired t-tests were then used to compare average performance before and after training completion.

The primary outcome measure for the study was self-selected comfortable walking speed, as measured by the 10-Meter Walk Test. Based on the population mean and standard deviation from two prior gait training studies with the GEMS-H device [14, 40], we estimated that a sample size of 46 would be needed to give approximately 80% power to detect a 0.14 m/s difference in self-selected comfortable walking speed between Pre and Post training at the 0.05 significance level. Only 41 subjects completed the protocol, resulting in approximately 76% power to detect the same 0.14 m/s difference in self-selected comfortable walking speed between Pre and Post training at the 0.05 significance level.

### Classification of evidence

This study provides Class III evidence that, for persons with sub-acute and chronic stroke, 18 45-minute sessions of gait training with the GEMS-H can lead to statistically significant improvements in walking speed, walking endurance, and balance.

## Results

Fifty-three participants were recruited to participate in the study. Due to a temporary suspension of research activities as a result of COVID-19, the 9 participants enrolled prior to March 2020 were withdrawn from the study. Once infection control policies were implemented and research activities resumed, 7 of the withdrawn participants were determined to be at a lower risk of infection according to Centers for Disease Control (CDC) guidance. These participants were re-enrolled and restarting the protocol from baseline. Over the course of the study, 12 participants were withdrawn for various reasons, as listed in Fig. 1. Of the remaining 41 participants, 39 completed all gait training and assessments, while 2 missed the one-month follow-up assessment due to COVID-19 concerns and moving out of the area.

### Clinical assessments

The relative changes of all clinical assessment scores at Mid, Post, and 1MFU compared to baseline (Pre) are presented in Table 2. For self-selected comfortable walking speed (10MWT - SSV), fastest walking speed (10MWT - FV), walking endurance (6MWT), and balance measures (BBS, FGA), all time points showed statistically significant improvements when compared to baseline performance. Additionally, there was a statistically significant improvement in motor recovery (Fugl-Meyer) and self-reported balance confidence (ABC) from Pre to Post and Pre to 1MFU. Statistically significant improvement in Stroke Specific Quality of Life was seen from Pre to Post, but no other changes were significant.

**Table 2:** Mean and standard deviation of the difference in clinical outcome measures relative to baseline performance

Outcome	Mid	Post	1MFU
10MWT – SSV, m/s	0.11 ± 0.14*	0.12 ± 0.15*	0.13 ± 0.16*
10MWT – FV, m/s	0.10 ± 0.13*	0.13 ± 0.15*	0.14 ± 0.19*
6MWT, m	32.86 ± 45.66*	44.19 ± 57.71*	47.39 ± 58.62*
BBS	1.56 ± 3.58*	2.34 ± 3.37*	2.93 ± 3.68*
FGA	1.88 ± 2.47*	2.02 ± 2.88*	1.80 ± 3.13*
5xSTS, s	-1.89 ± 4.56	-2.68 ± 6.68	-1.39 ± 7.51
Fugl-Meyer	2.32 ± 5.03	2.59 ± 5.74*	3.15 ± 6.57*
ABC	1.98 ± 13.49	4.59 ± 9.96*	4.35 ± 12.30*
SSQoL	6.09 ± 20.27	9.26 ± 13.68*	5.22 ± 18.87
SIS	0.57 ± 3.29	1.11 ± 3.84	1.49 ± 4.56
mFES	0.16 ± 1.13	0.00 ± 1.31	0.20 ± 1.52
PHQ-9	-0.68 ± 2.24	-0.50 ± 2.84	-0.19 ± 4.45

* Signifies statistically significant difference (*p* < 0.05) relative to baseline

Abbreviations: 10MWT-FV – 10-Meter Walk Test for fastest walking speed, 10MWT-SSV – 10-Meter Walk Test for self-selected comfortable walking speed, 5xSTS – 5 Times Sit-to-Stand Test, 6MWT – 6-Minute Walk Test, ABC – Activities-Specific Balance Confidence Scale, BBS – Berg Balance Scale, FGA – Functional Gait Assessment, mFES – modified Falls Efficacy Scale, PHQ-9 – Patient Health Questionnaire for depression, SIS – Stroke Impact Scale, SSQoL Stroke-Specific Quality of Life

Fifteen (37%) participants met or exceeded the MCID of 0.14 m/s for walking speed at Post with 12 (29%) carrying the improvement into the 1MFU. Similarly, 14 (34%) participants met or exceeded the MCID of 50 m for walking endurance at Post and 13 (32%) carried this improvement into the 1MFU. For the BBS, 12 (29%) and 10 (24%) participants met or exceeded the MDC of 4.13 points at Post and 1MFU, respectively. For the FGA, 15 (37%) participants met or exceed the MDC of 4.2 points at Post and 9 (22%) met or exceeded the MDC at the 1MFU.

### Device safety and immediate mobility assistance

Over the course of the intervention, the study cohort completed 738 training sessions (18 training sessions x 41 participants). Over the course of these training sessions, 34 adverse events occurred, producing an adverse event rate of 4.61%. Of these 34, only 6 occurred during training sessions and were determined to be possibly device related (hypertension, *n* = 1; bruising, *n* = 1; muscle cramp/fatigue, *n* = 2; fall, *n* = 1; knee pain, *n* = 1), leading to a device-related adverse event rate of 0.81%. For these 6 device-related events, 5 were anticipated while 1 (hypertension) was not anticipated. The outcome for all 6 device-related events was full recovery by the participant.

**Fig. 3:**
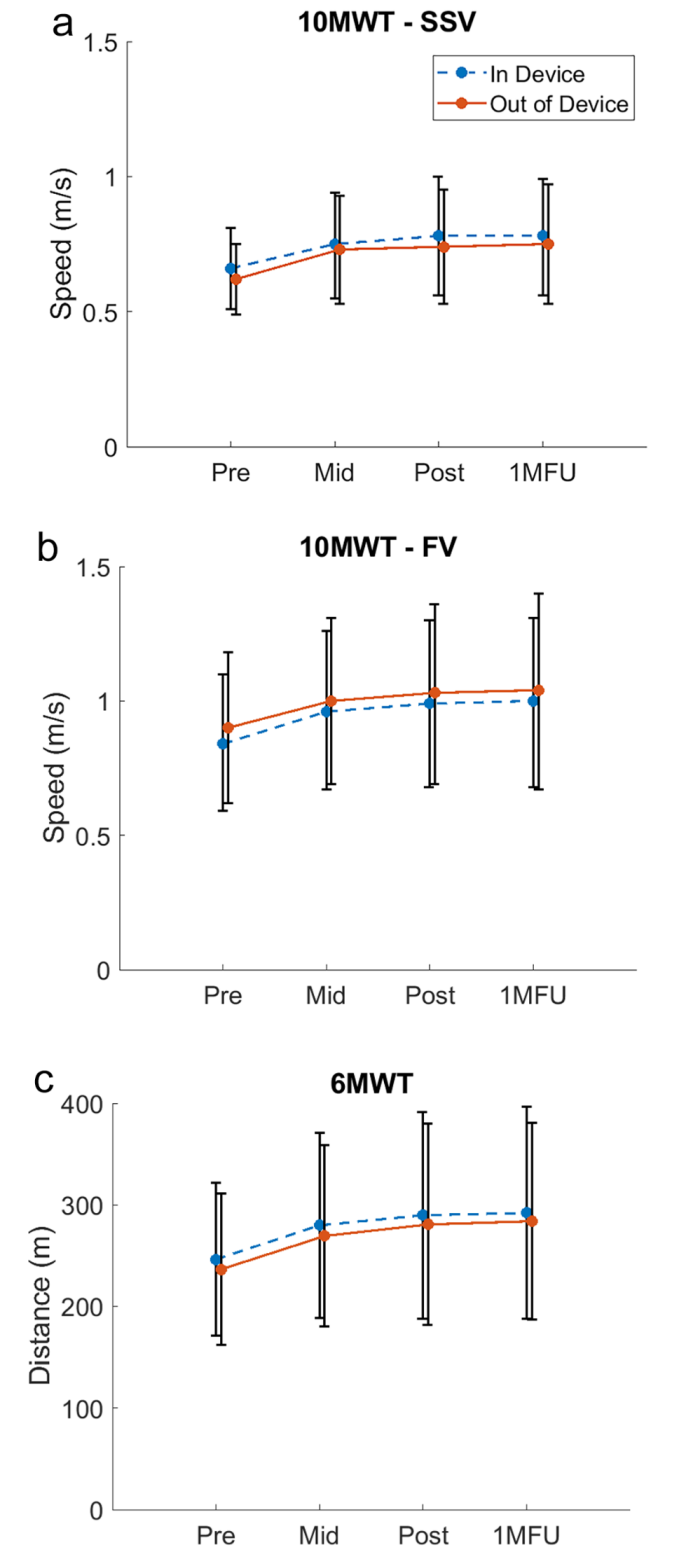
Mean and 95% confidence interval for: (**a**) self-selected comfortable walking speed, (**b**) fastest walking speed, and (**c**) walking endurance with and without the GEMS-H across assessment time points

Results for walking speed and endurance, both with and without the GEMS-H, across all four time points are illustrated in Fig. 3. On average, participants were able to increase their self-selected comfortable walking speed (10MWT - SSV) and walking endurance (6MWT) while in the device. Conversely, participants, on average, did not reach the same fastest walking speeds (10MWT - FV) with the device than without.

### Daily stepping activity

**Fig. 4:**
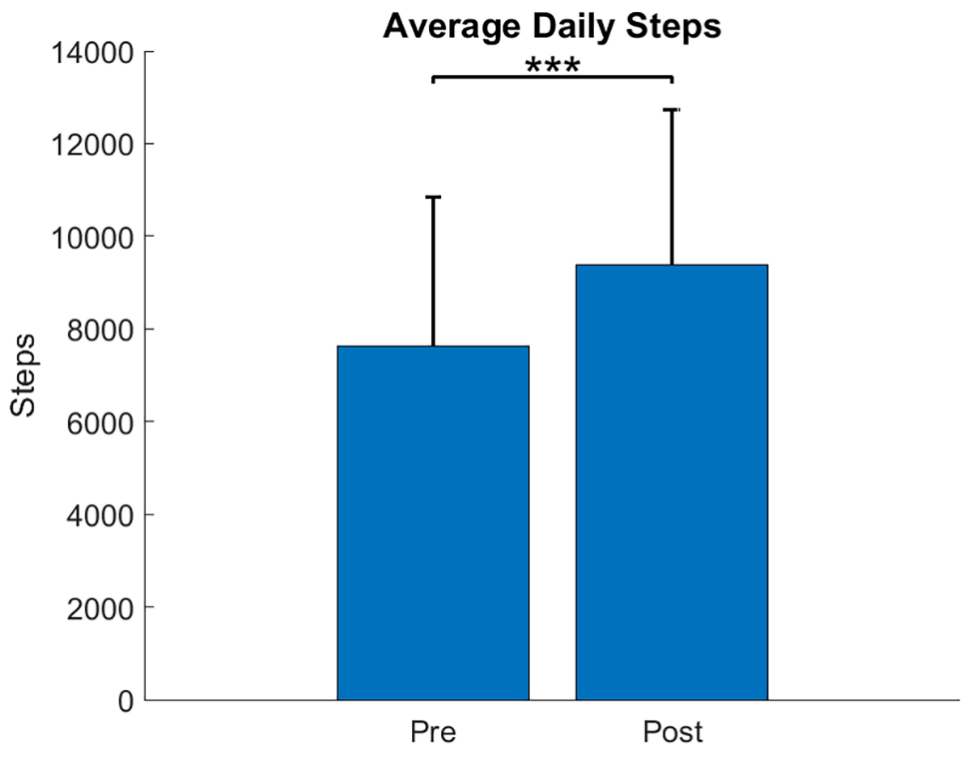
Average daily step count and 95% confidence interval before training with the GEMS-H (Pre) and after 18 training sessions (Post)

Of the 41 participants, 36 subjects’ data met the minimum wear time threshold of 33% and was included in the analysis. Figure 4 illustrates the average daily steps before and after training. On average, there was an increase of 1,750 steps per day (*p* < 0.001).

### Corticomotor excitability

Twenty-two individuals participated in TMS testing. Of these, 1 was unable to complete any testing sessions due to TMS intolerance, and 5 only completed a Pre session (unable to complete Post because of COVID-19 closure, *n* = 3; withdrawal from the study, *n* = 2). The remaining 16 individuals were included in the analysis.

Table 3 contains data from TMS testing at Pre and Post as well as the difference and *p*-value for each muscle. AMT significantly decreased in the paretic MG from 54.0% of the maximal stimulator output (MSO) at baseline to 48.3% MSO post training (*p* = 0.04). Six participants had emergence of paretic MEPs in at least one muscle from Pre to Post, indicating that the excitability of the ipsilesional hemisphere increased after training. This was particularly true for BF, which emerged in 4 participants from Pre to Post. At the Post assessment, prevalence of paretic MEPs was highest in the TA and RF (12 out of 16, 75%), followed by the BF (10 out of 16, 63%), and MG (8 out of 16, 50%). Prevalence of non-paretic MEPs was 100% for all muscles. There were no changes in MEP RMS, latency, or duration from Pre to Post for any muscle (*p* ≥ 0.08) while the silent period duration significantly increased in the non-paretic TA and MG (*p* ≤ 0.04).

**Table 3:** TMS outcomes [Mean (SD)] for all paretic and non-paretic muscles from pre, Post, the difference between assessments [Mean difference (95% CI)], and *p*-value

		Pre	Post	Post-Pre (95% CI)	*p*	
	**AMT (%MSO)**					
Paretic	TA	49.4 (13.8)	49.3 (12.5)	-0.08 (-2.8, 2.6)	0.95	
	MG	54.0 (15.6)	48.3 (14.3)	-5.7 (-10.8, 0.5)	0.04*	
	RF	54.8 (12.6)	57.1 (13.1)	2.3 (-2.4, 7.0)	0.30	
	BF	56.0 (10.3)	55.7 (16.1)	-0.3 (-12.5, 11.8)	0.95	
Non-Paretic	TA	40.3 (7.1)	40.2 (7.3)	-0.1 (-1.2, 1.0)	0.82	
	MG	45.6 (6.6)	44.9 (6.8)	-0.8 (-3.6, 2.1)	0.58	
	RF	42.1 (6.9)	41.8 (7.1)	-0.2 (-2.4, 1.9)	0.82	
	BF	43.0 (10.1)	42.6 (9.8)	-0.4 (-2.7, 1.8)	0.68	
	**MEP RMS/BEMG RMS**					
Paretic	TA	8.0 (5.7)	7.5 (4.8)	-0.5 (-2.6, 1.6)	0.61	
	MG	5.0 (2.5)	6.0 (3.3)	1.0 (-1.8, 3.8)	0.41	
	RF	4.5 (2.9)	4.8 (3.8)	0.3 (-2.2, 2.8)	0.80	
	BF	4.5 (1.6)	5.1 (3.7)	0.7 (-3.0, 4.3)	0.67	
Non-Paretic	TA	5.4 (3.2)	5.4 (2.5)	-0.03 (-1.2, 1.2)	0.95	
	MG	6.7 (2.1)	7.5 (3.3)	0.9 (-0.6, 2.3)	0.23	
	RF	8.5 (4.8)	7.7 (4.2)	-0.8 (-2.9, 1.3)	0.43	
	BF	5.1 (1.2)	5.4 (2.3)	0.3 (-0.6, 1.2)	0.52	
	**MEP Latency (ms)**					
Paretic	TA	37.0 (11.7)	35.8 (9.6)	-1.2 (-4.4, 2.0)	0.41	
	MG	33.1 (5.5)	33.3 (7.8)	0.2 (-3.5, 3.9)	0.88	
	RF	30.2 (7.5)	29.6 (9.7)	-0.6 (-4.9, 3.7)	0.76	
	BF	35.5 (15.6)	36.8 (13.7)	1.3 (-2.9, 5.5)	0.47	
Non-Paretic	TA	27.5 (4.3)	27.3 (4.3)	-0.2 (-1.8, 1.4)	0.79	
	MG	29.8 (3.6)	28.7 (3.2)	-1.0 (-2.3, 0.2)	0.09	
	RF	19.8 (4.0)	18.9 (3.6)	-0.9 (-2.0, 0.1)	0.08	
	BF	23.4 (3.5)	23.1 (3.1)	-0.3 (-1.7, 1.2)	0.72	
	**MEP Duration (ms)**					
Paretic	TA	52.4 (11.9)	54.5 (9.8)	2.1 (-7.1, 11.3)	0.62	
	MG	42.5 (9.1)	42.4 (6.8)	-0.1 (-7.5, 7.3)	0.97	
	RF	52.5 (13.5)	50.4 (10.1)	-2.1 (-11.0, 6.8)	0.61	
	BF	51.7 (10.2)	54.3 (13.0)	2.6 (-7.6, 12.7)	0.56	
Non-Paretic	TA	46.8 (12.5)	46.9 (10.5)	0.04 (-4.6, 4.7)	0.99	
	MG	41.6 (8.4)	44.7 (7.5)	3.1 (-2.2, 8.4)	0.23	
	RF	51.1 (11.2)	53.7 (8.5)	2.6 (-1.0, 6.2)	0.14	
	BF	48.8 (6.8)	48.5 (4.9)	-0.3 (-3.3, 2.7)	0.82	
	**Silent Period Duration (ms)**					
Paretic	TA	105.6 (46.9)	110.6 (27.9)	5.1 (-22.3, 32.6)	0.69	
	MG	48.3 (18.3)	133.9 (89.8)	85.7 (-178.1, 34.9)	0.30	
	RF	72.0 (28.6)	70.4 (15.9)	-1.6 (-23.9, 20.7)	0.87	
	BF	86.2 (65.4)	103.3 (48.5)	17.1 (-40.0, 74.2)	0.45	
Non-Paretic	TA	80.7 (30.1)	110.5 (43.8)	29.7 (1.9, 57.6)	0.04*	
	MG	85.5 (40.0)	111.4 (45.4)	25.9 (4.1, 47.8)	0.02*	
	RF	106.9 (46.1)	125.5 (35.2)	18.7 (-6.1, 43.4)	0.13	
	BF	96.9 (44.0)	115.6 (37.0)	18.7 (-9.0, 46.3)	0.17	

* Signifies statistically significant difference (*p* < 0.05)

Abbreviations: AMT – active motor threshold, BEMG – background EMG, BF – biceps femoris, CI – confidence interval, MEP – motor evoked potential, MG – medial gastrocnemius, MSO – maximal stimulator output, RF – rectus femoris, RMS – root mean squared value, TA – tibialis anterior

## Discussion

The objective of this study was to demonstrate that the Samsung Gait Enhancing and Motivating System-Hip (GEMS-H) Device is an effective gait rehabilitation tool for individuals with stroke and could safely be used in an outpatient setting.

### Clinical assessments

Based on the population of intended use (*n* = 41), self-selected comfortable walking speed, walking endurance, and balance (as measured by the 10MWT, 6MWT, BBS, and FGA) all statistically significantly improved after training with the GEMS-H (*p* < 0.001). While the group averages for these measures did not meet the corresponding MCIDs or MDCs, a number of individuals (≥ 29%) were able to show clinically significant improvement post-intervention. Additionally, a majority of those who did meet or exceed clinical thresholds at Post maintained these improvements at the 1MFU, event after training had stopped.

### Device safety, immediate mobility assistance, and daily stepping activity

Both the overall and device-related adverse event rates were below the threshold of 5% (4.61% and 0.81%, respectively). Additionally, all device-related adverse events resulted in full recovery by the participant, demonstrating that the GEMS-H device is safe to use in an outpatient clinical setting in patients with chronic stroke.

The group average increase in self-selected comfortable walking speed and walking endurance with the device compared to without suggests that wearers can easily acclimate to the device and immediate improvement can be seen for these outcomes, even on the first donning. The carryover of the reported trends at the 1MFU also suggests that participants were still able to demonstrate improved walking outcomes in the device, even after training had stopped. Additionally, the statistically significant increase in daily step count suggests that participants improved functional capacity in both the clinic and their day-to-day lives.

### Corticomotor excitability

We hypothesized that the training would induce an increase in corticomotor excitability of the legs because walking training represents a form of motor skill (re)training in individuals with stroke that likely elicits activity-dependent neuroplasticity [41, 42]. Previous studies have shown an increase in corticomotor excitability to the paretic leg after different types of walking training after stroke, which are associated with improvements in lower limb motor performance [43, 44]. The results from the current study suggest that training with the GEMS-H induced an increase in the corticomotor excitability for the paretic medial gastrocnemius as well as an increase in the level of intracortical inhibition in the contralesional hemisphere. After training, MEPs were able to be detected in 6 participants (35%) in muscles that had no MEPs before training. This emergence of MEPs also suggests that corticomotor excitability in the lesioned hemisphere was enhanced by the training, particularly in the lateral hamstring. Increases in the corticomotor excitability of the paretic medial gastrocnemius and lateral hamstring suggest that training may have a more pronounced effect on hip and knee flexors. Importantly, the use of the GEMS-H may enhance motor skill learning, and hence activity-dependent neuroplasticity, by adjusting the amount of assistance throughout training and enhancing the dosage and/or intensity of the walking intervention.

A leading model, the interhemispheric imbalance model [45], suggests that cortical excitability is imbalanced after stroke, with decreased excitability in the ipsilesional hemisphere and increased excitability in the contralesional hemisphere. Our results show evidence of increased excitability of the ipsilesional hemisphere and increased inhibition of the contralesional hemisphere, suggesting that training with the GEMS-H may help correct the interhemispheric imbalance after stroke.

### Limitations

Since this study was completed during the COVID-19 pandemic, there were certain limitations on how the training sessions were conducted. To observe appropriate physical distancing and infection control procedures, training sessions were restricted to small spaces, which limited the ability for subjects to maintain high intensities of training that may not be representative of the application of this device in a traditional clinical setting. Another limitation of COVID-19 was the sample size that was collected. With a temporary halt in the study at the start of the pandemic, enrollment numbers decreased in size and recruitment became especially difficult. When restarting recruitment, we were also selective about the individuals that could come in to be screened based on risk factors of developing severe COVID-19. After reopening, many potential participants had concerns about COVID-19 and did not feel safe coming into a hospital to participate in a clinical trial.

## Conclusions

This study reported on the safety and efficacy of gait training with the Samsung GEMS-H device in an outpatient clinical setting. Although adjustments to conditions and environment were made to accommodate COVID-19 policies, subjects were able to statistically improve walking speed, walking endurance, and balance after 18 training sessions with the GEMS-H. Additionally, a number of participants were able to obtain clinically significant improvements after training, with the majority maintaining said improvements after training had ended. Subjects also showed improved walking outcomes upon first donning while having a low adverse event rate of 4.61% and device-related adverse event rate of 0.81% throughout the course of the study. The improvement in average daily step counts as well as corticomotor excitability on the paretic side, suggests greater activity in day-to-day lives and enhanced activity-dependent neuroplasticity. These findings provide support for using the Samsung GEMS-H as a mobility assist device for gait training in the chronic stroke population.

## Data Availability

De-identified data are available from the authors upon reasonable request.

## Abbreviations

1MFU: 1-month follow-up after the last training session
10MWT: 10-Meter Walk Test
5xSTS: 5 Times Sit-to-Stand Test
6MWT: 6-Minute Walk Test
ABC: Activities-specific and Balance Confidence Scale
AD: Assistive devices
AFO: Ankle foot orthosis
AMT: Active motor threshold
BBS: Berg Balance Scale
BF: Biceps femoris
CDC: Centers for Disease Control
EMG: Electromyography
FGA: Functional Gait Assessment
FMA: Fugl-Meyer Assessment
FV: Fast walking velocity
GEMS-H: Samsung Gait Enhancing and Motivating System-Hip
MCID: Minimally clinically important difference
MDC: Minimal detectable change
MEP: Motor evoked potential
mFES: Modified Falls Efficacy Scale
MG: Medial gastrocnemius
Mid: After 9 training sessions
MSO: Maximal stimulator output
MVIC: Maximum voluntary isometric contraction
PHQ-9: Patient Health Questionnaire-9
Post: After 18 training sessions
Pre: Before training
RF: Rectus femoris
RMS: Root mean square
SIS: Stroke Impact Scale
SSQoL: Stroke Specific Quality of Life
SSV: Self-selected comfortable walking velocity
TA: Tibialis anterior
TMS: Transcranial magnetic stimulation
